# Supplementary material for: Predicting visual function by interpreting a neuronal wiring diagram
Source: Nature. 2024 Oct 2;634(8032):113–23. doi: 10.1038/s41586-024-07953-5 (PMC11446822; doi:10.1038/s41586-024-07953-5)
Supplement: Supplementary file 6 — CRF and ERF predictions for individual TmY4 and TmY9 cells. Analogous to Supplementary Data 3, but for TmY target types. Shown are the top four monosynaptic pathways, the strongest pathway passing through each of the top ten intermediary types (ranking from Extended Data Fig. 7), and the trisynaptic pathway Tm1–TmY–Dm3–TmY (see the section entitled Prediction of spatial normalization). [file 41586_2024_7953_MOESM6_ESM.zip › DataS4/TmY9qΓèÑ/720575940605280614.pdf]

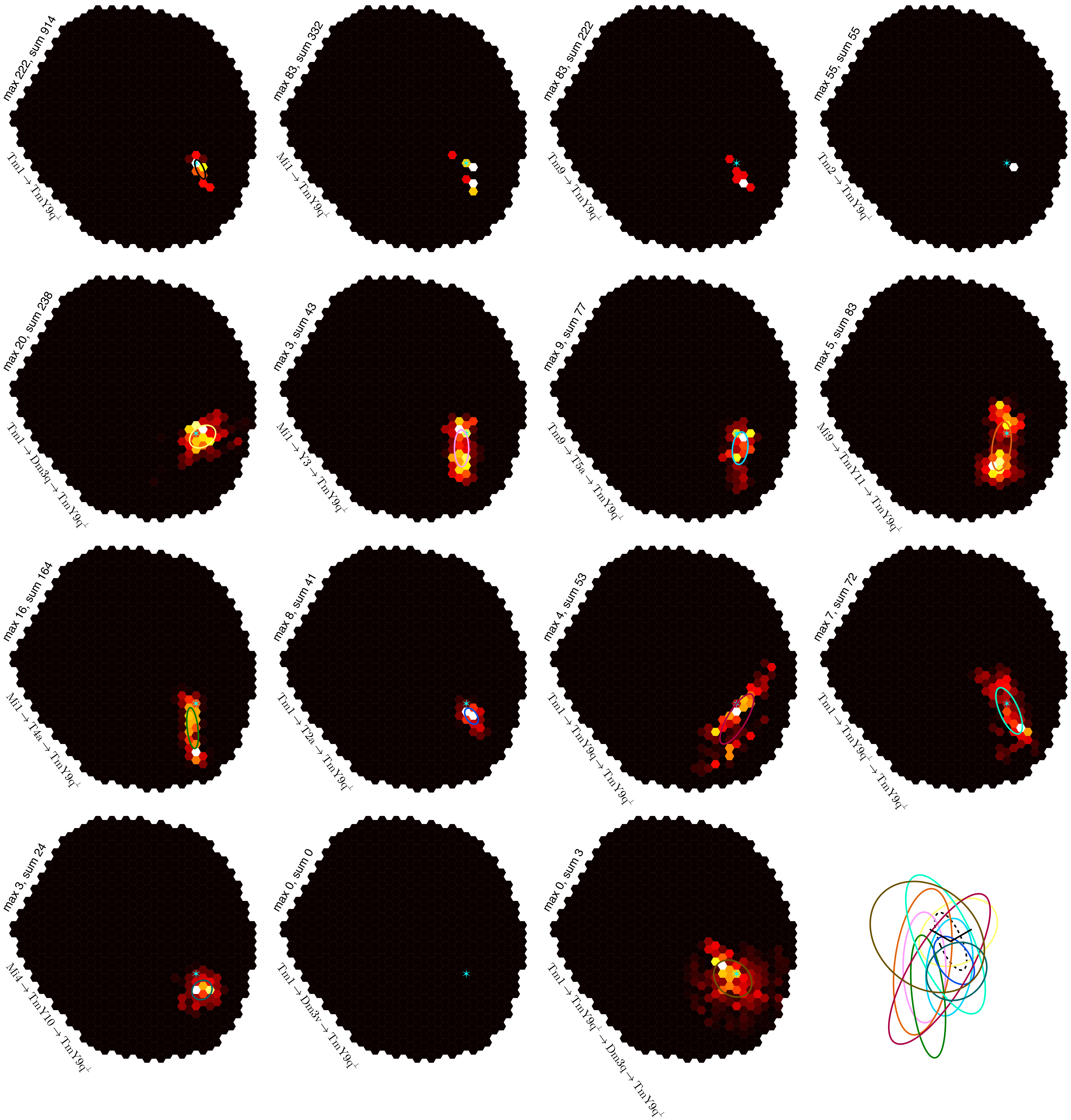

max 222, sum 914
Tm1 → TmY9q⊥
max 83, sum 332
Mi1 → TmY9q⊥
max 83, sum 222
Tm9 → TmY9q⊥
max 55, sum 55
Tm2 → TmY9q⊥
max 20, sum 238
Tm1 → Dm3q → TmY9q⊥
max 3, sum 43
Mi1 → Y3 → TmY9q⊥
max 9, sum 77
Tm9 → T5a → TmY9q⊥
max 5, sum 83
Mi9 → TmY11 → TmY9q⊥
max 16, sum 164
Mi1 → T4a → TmY9q⊥
max 8, sum 41
Tm1 → T2a → TmY9q⊥
max 4, sum 53
Tm1 → TmY9q⊥ → TmY9q⊥
max 7, sum 72
Tm1 → TmY9q⊥ → TmY9q⊥
max 3, sum 24
Mi4 → TmY10 → TmY9q⊥
max 0, sum 0
Tm1 → Dm3v → TmY9q⊥
max 0, sum 3
Tm1 → TmY9q⊥ → Dm3q → TmY9q⊥
